# Supplementary material for: Optogenetic manipulation of a value-coding pathway from the primate caudate tail facilitates saccadic gaze shift
Source: Nat Commun. 2020 Apr 20;11:1876. doi: 10.1038/s41467-020-15802-y (PMC7171130; doi:10.1038/s41467-020-15802-y)
Supplement: Supplementary file 1 — Supplementary Information [file 41467_2020_15802_MOESM1_ESM.pdf]

Supplementary Information

Optogenetic manipulation of a value-coding pathway from the primate caudate tail facilitates saccadic gaze shift

Amita et al.

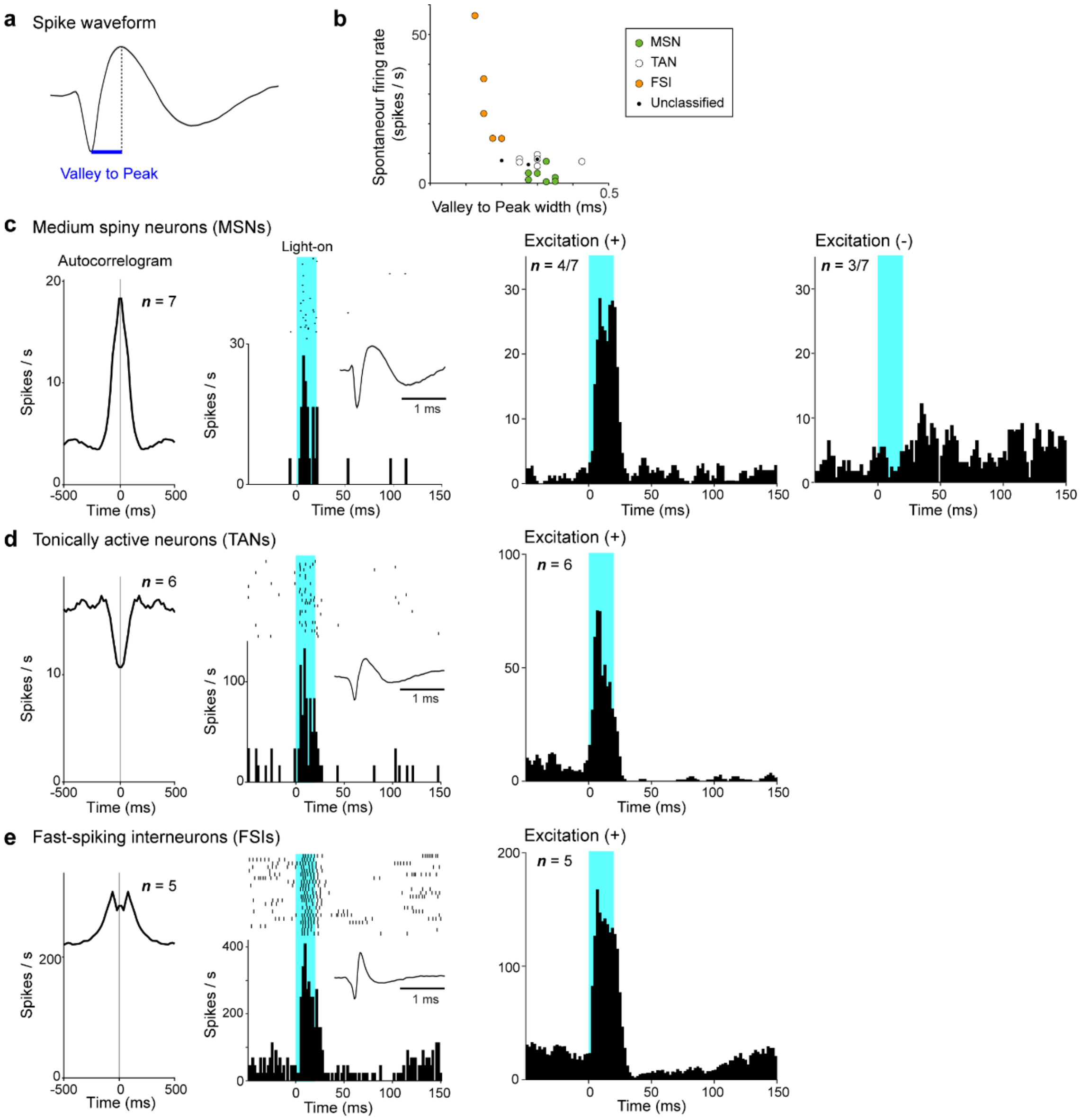

Supplementary Figure 1. Diverse CDt neuron types showed excitatory responses to optical stimulation.

(**a**) Quantification of spike waveform width. (**b**) Classification of CDt neuronal types with spike features (Spontaneous firing rate vs. Valley to Peak width). Green circles, open squares, and orange triangles indicate 7 MSNs, 6 TANs, 5 FSIs and 3 unclassified neurons, respectively. (**c**) The average autocorrelogram of 7 MSNs (left). Response of a representative MSN (middle-left) and averaged response of MSNs (middle-right: Excitation (+); 4 out of 7 neurons) showing significant excitatory response to optical stimulation ($P < 0.05$, Wilcoxon singed-rank test, two-sided). Average response of MSNs (right: Excitation (-); 3 out of 7 neurons) showing no excitatory response to the stimulation.

Insets show spike waveforms. (**d**) The average autocorrelogram of 6 TANs (left). Response of a representative TAN (middle) and averaged response of TANs (right: Excitation (+); 6 out of 6 neurons) to optical stimulation. (**e**) The average autocorrelogram of 5 FSIs (left). Response of a representative FSI (middle) and averaged response of FSIs (right: Excitation (+); 5 out of 5 neurons) to optical stimulation.

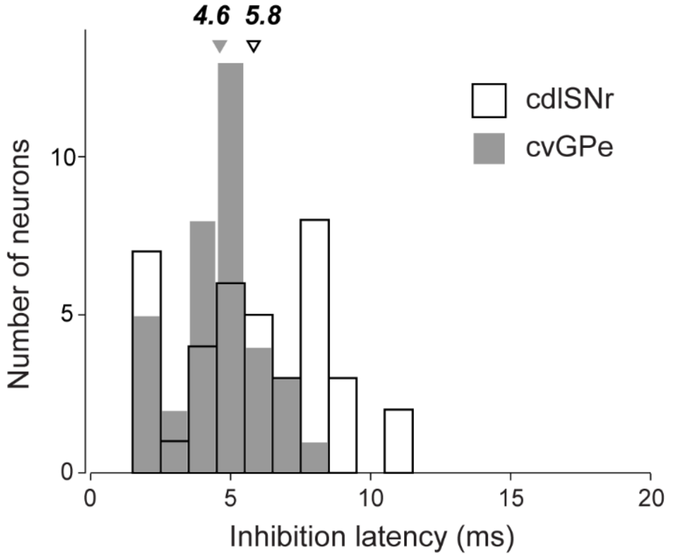

Supplementary Figure 2. Latency of responses in cdlSNr and cvGPe to optical stimulation.

Response latencies of cdlSNr (*n*=39 neurons, white histograms) and cvGPe neurons (*n*=36 neurons, gray histograms) to the stimulation. White and gray inverted triangles indicate the average response latencies of cdlSNr and cvGPe, respectively.

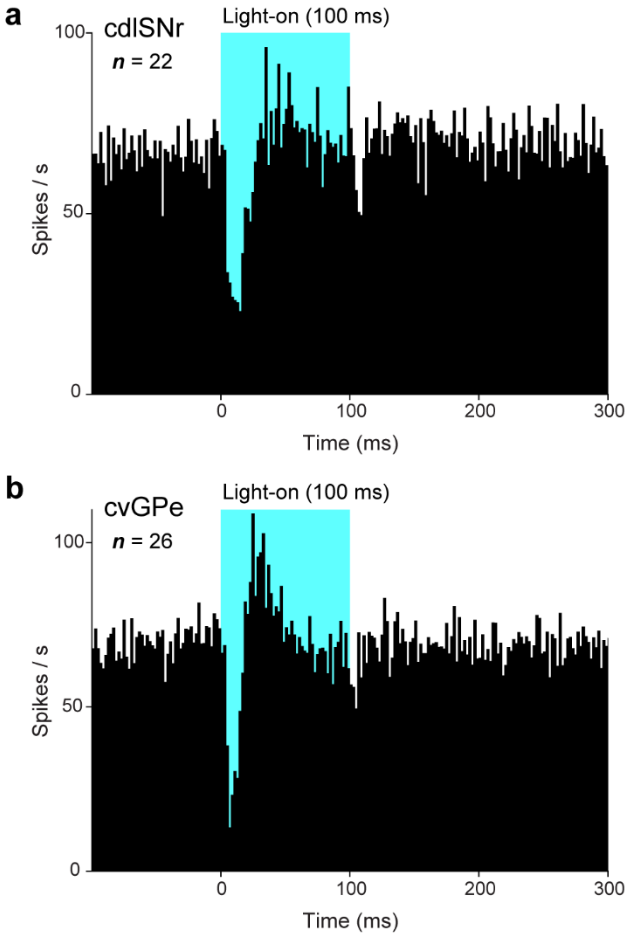

Supplementary Figure 3. Responses of cdlSNr and cvGPe neurons to longer optical stimulation (100 ms).

(**a**) Averaged response of 22 cdlSNr neurons to 100 ms of optical stimulation to CDt-cdlSNr pathway. (**b**) Averaged response of 26 cvGPe neurons to 100 ms of optical stimulation to CDt-cvGPe pathway.

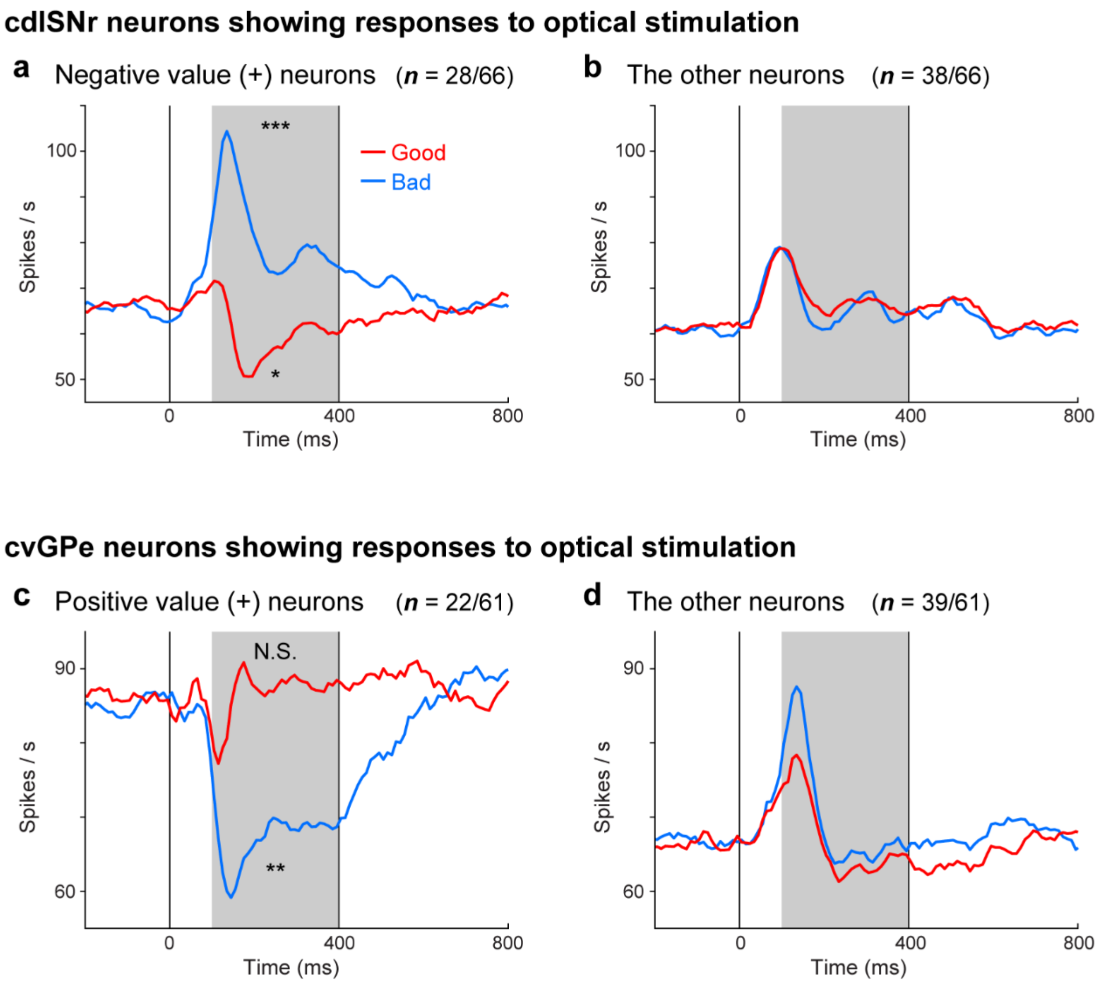

Supplementary Figure 4. Classified populations of value-coding neurons showing responses to optical stimulation in cdlSNr (top panels) and cvGPe (bottom panels). (**a**) Averaged response of 28 negative value-coding cdlSNr neurons to good objects (red) and bad objects (blue). These 28 neurons showed significant inhibitory responses to good objects (P = 0.026, Wilcoxon signed-rank test, two-sided) and significant excitatory responses to bad objects ($P = 2.1 \times 10^{-6}$, Wilcoxon signed-rank test, two-sided). The response to good or bad objects in a test window (light-gray shade; 100-300 ms after the object onset) was statistically compared with a baseline window (0-300 ms before the object onset). *, *** indicates $P < 0.05$, 0.001. (**b**) Averaged response of 38 non-negative value-coding cdlSNr neurons to good objects (red) and bad objects (blue). (**c**) Averaged response of 22 positive value-coding cvGPe neurons to good objects (red) and bad objects (blue). These 22 neurons showed significant inhibitory responses to bad objects (P = 0.0024, Wilcoxon signed-rank test, two-sided) but not to good objects (P = 0.41, Wilcoxon signed-rank test, two-sided). Light-gray shade shows the test window. N.S. indicates $P > 0.05$. ** indicates $P < 0.01$. (**d**) Averaged response of 39 non-positive value-

coding cvGPe neurons to good objects (red) and bad objects (blue).

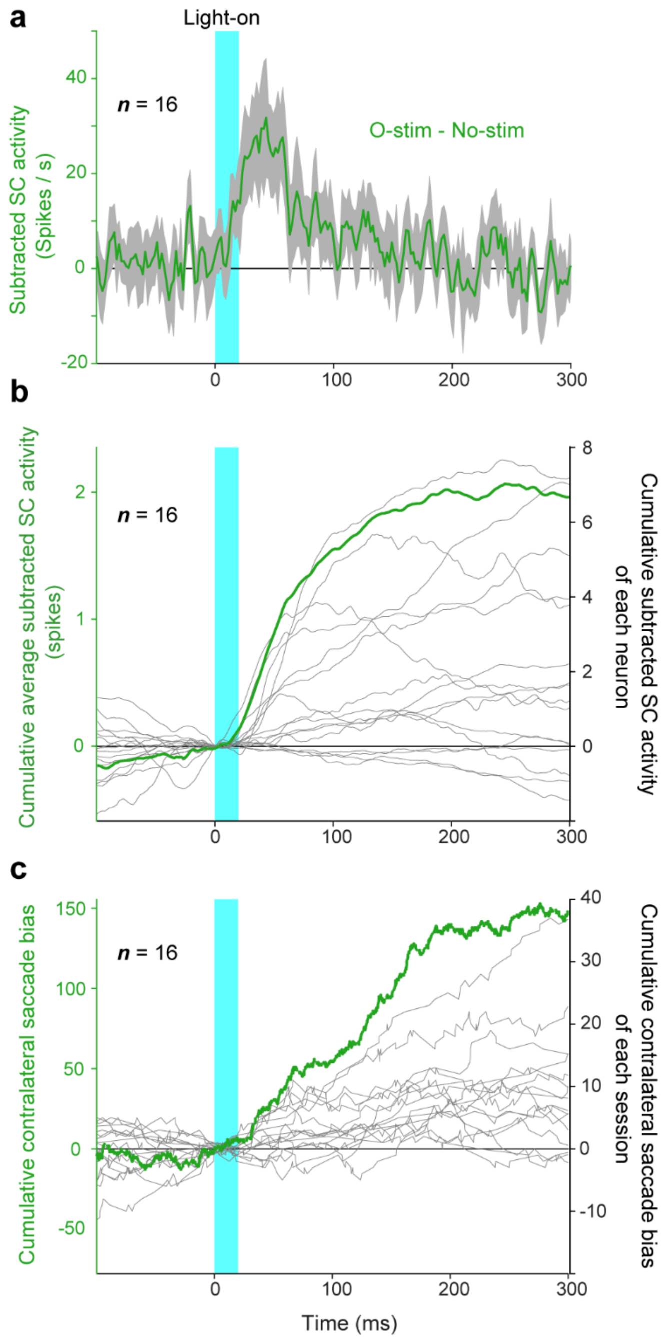

Supplementary Figure 5. Response of SC to optical stimulation of CDt-cdlSNr pathway.

(**a**) Average subtracted activity (stimulation trials minus no-stimulation trials) of 16 SC neurons during optical stimulation to CDt-cdlSNr pathway. Green line and gray shade indicate mean and SEM, respectively. (**b**) The cumulative plot of average subtracted SC activity (green line) showed prolonged response after the stimulation. Each gray line indicates cumulative subtracted SC activity of each neuron (*n*=16). (**c**) The cumulative

plot of contralateral saccade bias (green line) also showed prolonged increase after the stimulation. Each gray line indicates cumulative saccade bias of each session (n=16). The data show the stimulation effect on saccade during the same sessions in Supplementary Fig. 4a, b.

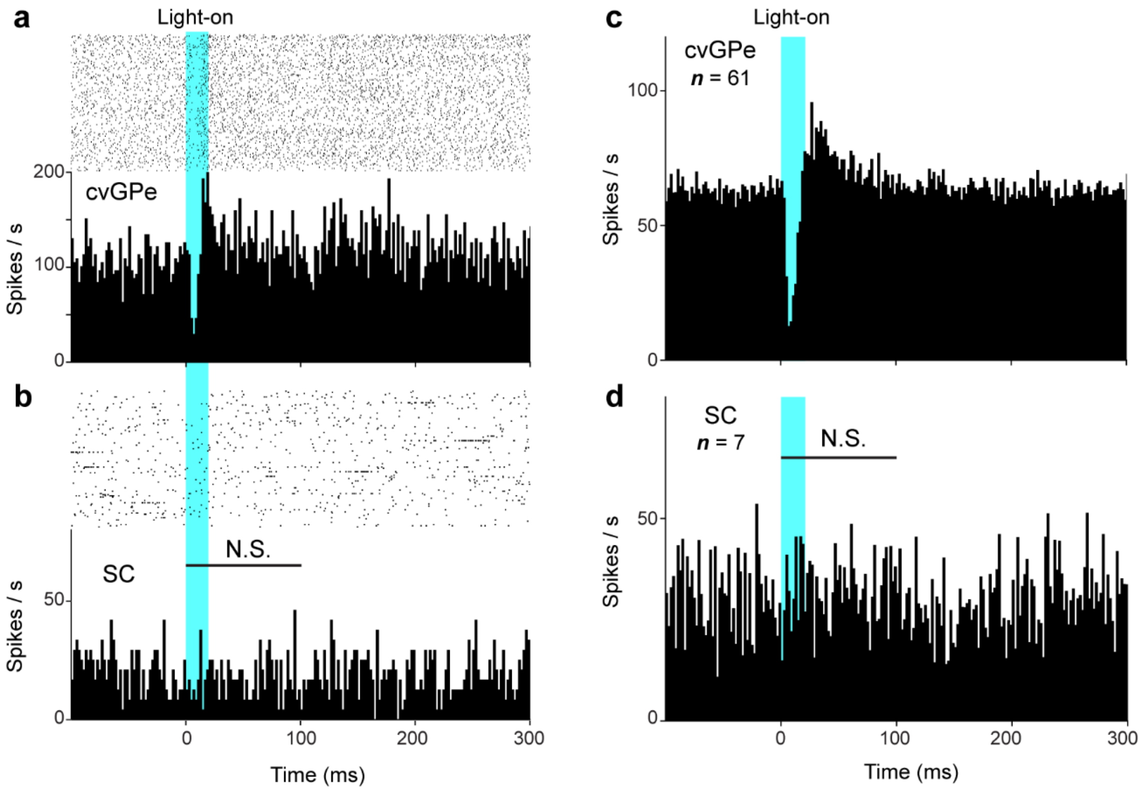

Supplementary Figure 6. SC response to optical stimulation of the CDt-cvGPe pathway.

(**a**) A representative cvGPe neuron that was significantly inhibited by the optical stimulation (P = 1.5 × 10^{-8}, Wilcoxon signed-rank test, two-sided). (**b**) A representative SC neuron that was not significantly modulated during the optical stimulation (Supplementary Fig. 5a) (P = 0.46, Wilcoxon signed-rank test, two-sided). N.S. indicates P > 0.05. (**c**) Averaged activity of 61 cvGPe neurons showing significant responses to the stimulation. Same data as Fig. 4d. (**d**) Averaged activity of seven SC neurons during optical stimulation to the CDt-cvGPe pathway. The population activity of SC neurons did not show notable modulation by the stimulation (P = 0.58, Wilcoxon signed-rank test, two-sided). N.S. indicates P > 0.05.

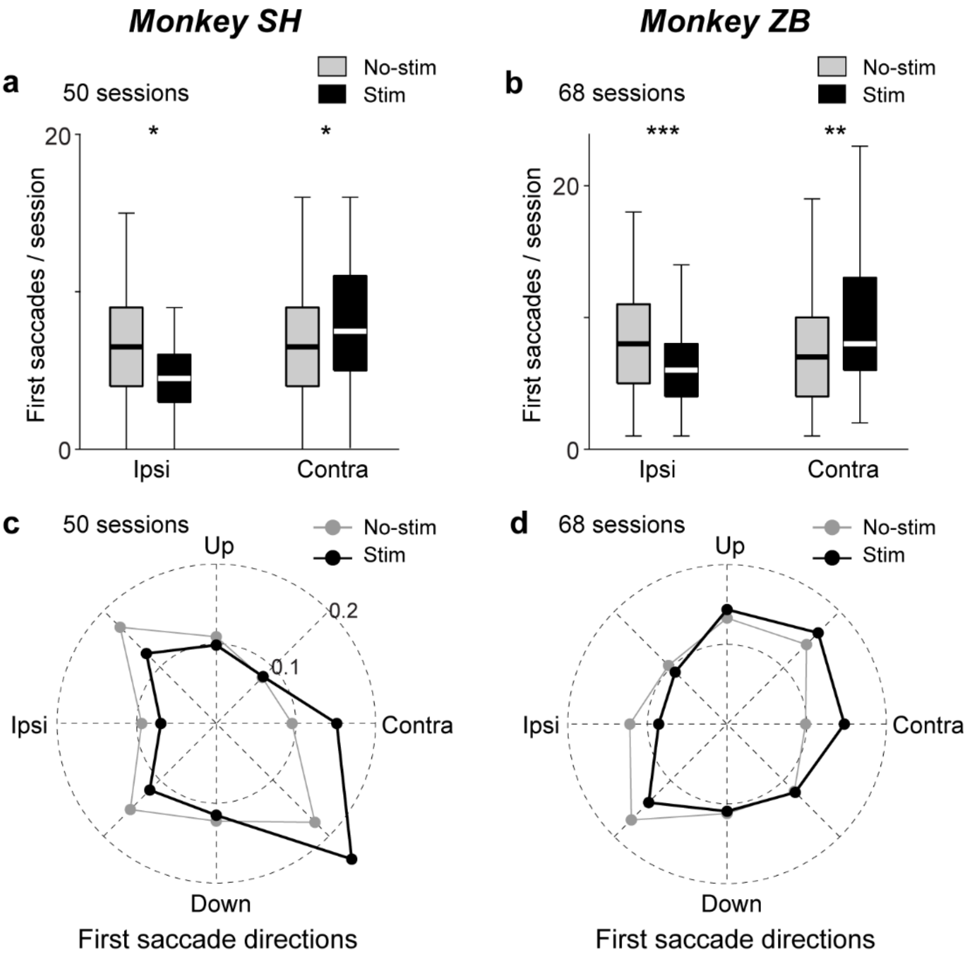

Supplementary Figure 7. First saccade bias by optical stimulation of CDt-cdlSNr pathway.

(**a**) The number of first saccades per session in 0-200 ms after no-stimulation (gray) vs. stimulation (black) of the CDt-cdlSNr pathway. In monkey SH, ipsilateral saccades significantly decreased after stimulation (n=50, P = 0.010, Wilcoxon singed-rank test, two-sided), while contralateral saccades significantly increased (n=50, P = 0.010, Wilcoxon singed-rank test, two-sided). The band indicates the median, the box indicates the first and third quartiles and the whiskers indicate ± 1.5 × interquartile range. * indicates $P < 0.05$. (**b**) In monkey ZB, ipsilateral saccades significantly decreased after stimulation (n=68, $P < 0.001$, Wilcoxon singed-rank test, two-sided), while contralateral saccades significantly increased (n=68, P = 0.0015, Wilcoxon singed-rank test, two-sided). Same format as Supplementary Fig. 7a. **, *** indicate $P < 0.01, 0.001$. (**c**) Mean direction of first saccades within 200 ms after the optical stimulation in monkey SH. The subject made more saccades to the lower contralateral quadrant in stimulation trials (black) than in no-stimulation trials (gray). Same format as Fig. 6h. (**d**) Mean direction of

first saccades within 200 ms after the optical stimulation in monkey ZB. The subject made more saccades to the upper contralateral quadrant in stimulation trials (black) than no-stimulation trials (gray).

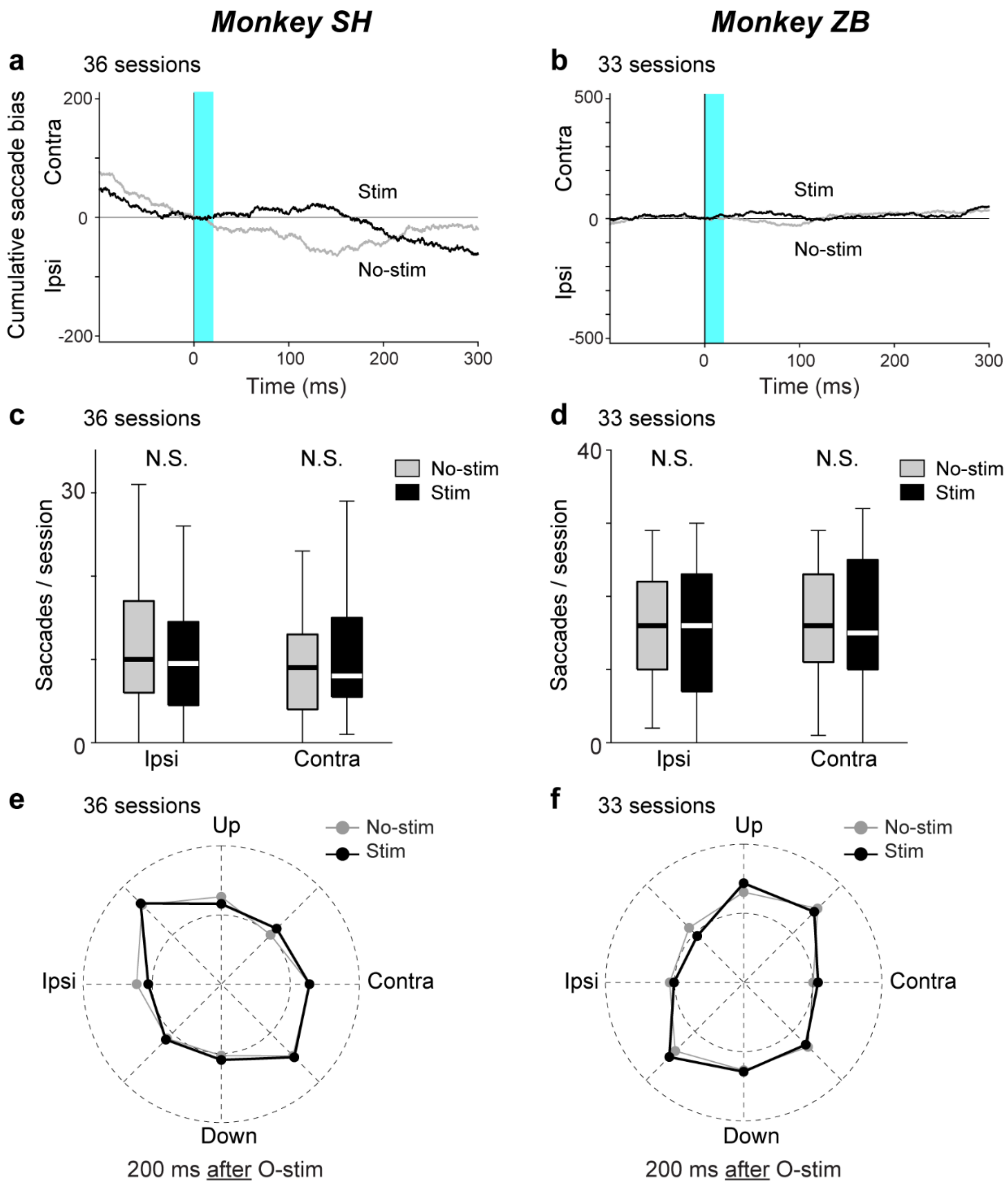

Supplementary Figure 8. No effect of optical stimulation of CDt-cvGPe pathway on saccades.

(**a**) Temporal dynamics of the cumulative saccade bias from the stimulation onset in monkey SH (total 36 sessions). Same format as Fig. 6d. (**b**) Temporal dynamics of the cumulative saccade bias from the stimulation onset in monkey ZB (total 33 sessions). (**c**) The number of saccades per session in 0-200 ms after no-stimulation (gray) vs. stimulation (black) of the CDt-cvGPe pathway. In monkey SH, neither ipsilateral saccade (n=36, P = 0.55, Wilcoxon singed-rank test, two-sided) nor contralateral saccade (n=36, P = 0.62, Wilcoxon singed-rank test, two-sided) was significantly modulated after the

stimulation. The band indicates the median, the box indicates the first and third quartiles and the whiskers indicate ± 1.5 × interquartile range. N.S. indicates P > 0.05. (**d**) In monkey ZB, neither ipsilateral saccade (n=33, P = 0.50, Wilcoxon singed-rank test, two-sided) nor contralateral saccade (n=33, P = 0.27, Wilcoxon singed-rank test, two-sided) was significantly modulated after the stimulation. Same format as Supplementary Fig. 8a. N.S. indicates P > 0.05. (**e**) Mean direction of first saccades within 200 ms after the optical stimulation in monkey SH. Same format as Fig. 6h. (**f**) Mean direction of first saccades within 200 ms after the optical stimulation in monkey ZB.
